# Colposcopy “Under the Loupe”: Diagnostic Accuracy of Colposcopic Examination of CIN2+ Lesions: Systematic Review and Meta-Analysis of 141,355 Cases

**DOI:** 10.3390/cancers18142239

**Published:** 2026-07-13

**Authors:** Marco Cerbone, Rosa De Vincenzo, Alessia Auriola, Miriam Dellino, Eliano Cascardi, Carmine Carriero, Vincenzo Pinto, Mauro Francesco Pio Maiorano, Giorgio Maria Baldini, Caterina Ricci, Maria Teresa Evangelista, Vera Loizzi, Gennaro Cormio, Ettore Cicinelli

**Affiliations:** 1 First Unit of Obstetrics and Gynecology, Department of Interdisciplinary Medicine (DIM), University of Bari “Aldo Moro”, Policlinico of Bari, 70124 Bari, Italygennaro.cormio@uniba.it (G.C.);; 2 Dipartimento Ostetricia e Ginecologia, Università Cattolica del Sacro Cuore, 00168 Rome, Italy; 3 Dipartimento Scienze Della Salute Della Donna, del Bambino e di Sanità Pubblica, Fondazione Policlinico Universitario Agostino Gemelli IRCCS, 00168 Rome, Italy; 4 Unit of Gynaecology and Obstetrics, Department of Woman, Child and General and Specialized Surgery, University of Campania “Luigi Vanvitelli”, 80168 Naples, Italy; 5 Pathology Unit, Department of Precision and Regenerative Medicine and Ionian Area (DiMePRe-J), University of Bari “Aldo Moro”, Piazza Giulio Cesare 11, 70124 Bari, Italy; 6 Clinicalized Gynecological Oncology Unit, IRCCS Istituto Tumori ‘Giovanni Paolo II’, Viale Orazio Flacco 65, 70124 Bari, Italy; 7 Department of Translational Biomedicine and Neuroscience, University of Bari “Aldo Moro”, 70124 Bari, Italy

**Keywords:** colposcopy, diagnostic accuracy, sensitivity, specificity, ROC curve, metanalysis, systematic review, CIN2, CIN3, CIN2+

## Abstract

**Background**: Colposcopy is a widely used clinical and diagnostic tool for detecting cervical intraepithelial neoplasia grade 2 or higher (CIN2+). This paper aims to analyze the diagnostic accuracy, pooled sensitivity, specificity, likelihood ratios, and overall diagnostic performance of colposcopy. **Methods**: A systematic review of papers on diagnostic accuracy, preregistered in PROSPERO (CRD420251028776), was conducted following PRISMA-DTA guidelines. Statistical analyses were performed using SPSS 29 and METADISC 2.0. Study quality and risk of bias were assessed using QUADAS-2. **Results**: A total of 49 studies, including 141,355 colposcopic examinations, were included in the final analysis. The overall sensitivity was 0.745 (95% CI: 0.684–0.797) and specificity was 0.831 (95% CI: 0.770–0.879). The diagnostic odds ratio was 14.388 (95% CI: 9.673–21.400). The positive likelihood ratio was 4.419 (95% CI: 3.260–5.991). Conversely, the negative likelihood ratio was 0.307 (95% CI: 0.249–0.378). The area under the curve was 0.8569. Significant heterogeneity was observed across studies (I^2^ > 93.2%). **Conclusions**: Colposcopy demonstrates moderate-to-high diagnostic accuracy in detecting CIN2+ lesions, with a good balance between sensitivity and specificity. However, significant variability exists among studies, highlighting the need for the implementation of standardized colposcopic criteria, improved examiner training, and adjunctive diagnostic methods such as HPV testing and ancillary techniques to enhance accuracy and reduce false positives and false negatives. Future research should focus on minimizing interobserver variability and refining diagnostic algorithms to optimize colposcopic performance.

## Simple Summary

This systematic review and meta-analysis evaluated the diagnostic accuracy of colposcopy for detecting cervical intraepithelial neoplasia grade 2 or worse (CIN2+). A total of 49 studies, including 141,355 colposcopic impressions, were analyzed. Overall, colposcopy demonstrated good diagnostic performance, with a pooled sensitivity of 74.5% and specificity of 83.1%, indicating a balanced ability to correctly identify both diseased and non-diseased cases. Despite these encouraging results, substantial heterogeneity was observed across studies. The findings suggest that while colposcopy remains an effective diagnostic tool, its accuracy could be improved through standardized assessment criteria, enhanced examiner training, and the integration of complementary diagnostic methods such as HPV testing.

## 1. Introduction

Cervical cancer (CC) was responsible for 662,044 cases and 348,709 deaths in 2022, corresponding to the fourth leading cause of cancer morbidity and mortality in women worldwide [1]. CC has the highest incidence rates in Africa (Eastern, Southern, and Middle 40.42, 34.89, and 31.12 cases per 100,000, respectively), and Melanesia (27.59 per 100,000). The highest country-specific age-standardized incidence rate were recorded in Eswatini, Zambia, and Malawi (95.9, 71.5, and 70.9 cases per 100,000, respectively), countries with high AIDS prevalence [2]. CC is a preventable disease primarily caused by persistent infection with high-risk human papillomavirus (HPV) types, which lead to cell-cycle dysfunction and cancer development, with a disease pathway progressing from HPV infection through precancerous stages to invasive disease [3]. Understanding the natural history of HPV has been essential for comprehending the progression of CC and has led to the development of worldwide screening strategies. The evidence-based CC screening guidelines are different and based substantially on four tools (HPV-DNA testing, Pap test, HPV/Pap cotest and visual inspection with acid), as well as to select women at high-risk of CC for referral to second-level examination, that is, colposcopic examination [4]. Colposcopic examination of the cervix enables direct and magnified visualization of cervical abnormalities and, with the application of acetic acid and Lugol’s solution, enables the differential diagnosis of a wide range of cervical clinical presentations, ranging from benign, low-grade, and high-grade lesions. In the latter cases, colposcopists can perform targeted biopsies to obtain the histopathological confirmation and to guide the appropriate management and treatment of the CIN2+ disease.

## 2. Study Aims

The diagnostic accuracy of colposcopy in detecting CIN2+ lesions has been previously examined and is considered to have high specificity but low-to-moderate sensitivity [5,6,7,8]. Several factors influence the diagnostic accuracy of the examination, including examiner expertise, variations in subjective colposcopic interpretation, and differences in lesion characteristics. Moreover, the clinician’s pre-test knowledge of the patient’s clinical history (including risk factors such as smoking, previous infection, HPV vaccination status, parity, sexual habits, HPV-DNA test and Pap test results) is crucial for the interpretation of colposcopic pictures. Most of the previous studies exhibit high heterogeneity in study population, raising concerns about the estimation of diagnostic accuracy of colposcopy in detecting CIN2+ lesions and highlighting in the need for a systematic evaluation of the literature and the execution of meta-analysis and meta-regressions for moderators. Understanding the strengths and limitations of the colposcopic impression is essential to optimize cervical-cancer-screening strategies and integrate complementary diagnostic approaches to enhance CIN2+ early detection and treatment outcomes.

This study aims to assess the pooled diagnostic accuracy of colposcopy in detecting CIN2+ lesions of the cervix by calculating overall sensitivity, specificity, positive and negative likelihood ratios, diagnostic odds ratios, and the summary receiver operating characteristic (sROC) curve. Additionally, meta-regressions analysis for moderators (country income, type of study, HPV pre-test knowledge) were performed to evaluate the effects of potential sources of variability, including threshold effects and to provide insights into optimizing colposcopy as a diagnostic tool.

## 3. Materials and Methods

### 3.1. Methods of the Systematic Review

A systematic review, preregistered in PROSPERO https://www.crd.york.ac.uk/PROSPERO/view/CRD420251028776 (accessed on 12 May 2026), was conducted according to the PRISMA-DTA (Preferred Reporting-Items for Systematic-Reviews and Meta-Analyses) guidelines. A comprehensive literature review was performed in MEDLINE, PubMed, Scopus, and Google Scholar, using the keywords cervix, cervical cancer, colposcopy, diagnostic accuracy, sensitivity, and specificity. Two independent reviewers screened the retrieved studies based on predefined inclusion and exclusion criteria using the freeware version of the web app of Rayyan software © vers 4.1.3 (Qatar Computing Research Institute, Doha, Qatar, 2025, http://rayyan.qcri.org) [9]. The PRISMA checklist can be found in the Supplementary Materials [10].

The index test of this study was the impression of a CIN2+ lesion at colposcopic examination, considering as a threshold any HSIL at colposcopic impression deemed worthy of biopsy. In cases of tests with multiple colposcopic impressions according to different classification systems, we considered the IFCPC guidelines and nomenclature as the gold standard. The reference standard of this study was the confirmation of CIN2+ lesions of the cervix at histopathological examination. Inclusion criteria encompassed original research articles reporting diagnostic accuracy data on the target condition of CIN2+ lesions of the cervix. including a 2 × 2 table with true positives, true negatives, false positives, and false negatives. In selected cases, the metrics were reversely extracted from data on diagnostic accuracy, sensitivity, specificity, and study population. In the case of systematic reviews on the topic, the cited literature was retrieved, and the articles were extracted and reviewed manually. Studies were manually excluded if they were duplicated, lacked retrievable full-text data, were not in English to ensure the correct understanding of the data, were case reports or case–control studies, or did not contain relevant diagnostic accuracy metrics. Moreover, we excluded publications on PAP SMEAR, HPV test and visual inspection with acid accuracy alone, and cervical direct photography or multispectral colposcopy. Any article involving the use of any artificial intelligence (IA) technology for the evaluation of colposcopic images was excluded [11]. We performed a grey literature search on the main generic search engine, but no data with available metrics were retrieved. Inclusion and exclusion criteria and data extraction were applied manually, without any artificial intelligence software.

### 3.2. Methods of the Meta-Analysis

Data extraction included details on sample size, unit of analysis (by woman vs. by exam), study design (prospective vs. retrospective), patient characteristics, country and economic status (low–middle income countries (LMICs) vs. high-income countries (HICs), clinical indication for the colposcopic exam, knowledge of pre-test HPV test by the operator, colposcopic assessment methods and data on the diagnostic accuracy of selected studies [12]. Statistical analyses were performed using Microsoft Windows Excel © (Microsoft Corporation, San Diego, CA, USA, 2023) and Statistical Package for Social Sciences, SPSS © vers. 29 (IBM, San Francisco, CA, USA, 2023), while MetaDisc 2.0 © https://ciberisciii.shinyapps.io/MetaDiSc2/ (accessed on 12 May 2026) with R packages glmer, lme4, msm, meta, ggplot2, polyarea and plotly was used to generate summary tables and perform meta-analytical calculations and meta-regressions [13,14]. Mean age was computed by SPSS. All meta-analysis statistics were conducted using a bivariate random-effect model to account for pooled sensitivity, specificity, positive and negative likelihood ratios (PLR and NLR), and diagnostic odds ratios (DORs), with a 95% confidence interval (CI). Heterogeneity was assessed using Cochran’s Q statistic and quantified with the I^2^ statistic, with values above 75% considered indicative of substantial heterogeneity. To evaluate the overall diagnostic performance of the test, a summary receiver operating characteristic (sROC) curve was generated. Statistical significance was defined as *p* < 0.05. We conducted meta regressions for the following moderators: knowledge of HPV pre-test, (pre-HPV triage vs. HPV-primary), type of study and country income level. A leave-out analysis was performed to assess the potential influence of high bias studies on the overall results. The exclusion criteria were studies with a high risk of bias based on quality assessment, older studies that may have been affected by temporal bias effects, large cohort studies with potential influence on pooled estimates, and studies using spectral analysis, which may have introduced methodological heterogeneity. Since the number of studies within each individual category was limited and unbalanced, separate analyses for each exclusion criteria were not considered appropriate. Therefore, these studies were grouped into a single leave-out analysis to provide a more consistent evaluation of their overall impact on the findings.

### 3.3. Methods of the Assessment of Risk of Bias

The overall risk-of-bias (RoB) was evaluated using the QUADAS-2 (Quality Assessment of Diagnostic Accuracy Studies) tool and visualized via RobVis tool vers 0.2.9 https://mcguinlu.shinyapps.io/robvis/ (accessed on 12 May 2026) by three independent reviewers in a double-blind manner. The four domains evaluated were: patient selection, index test, reference standard, and flow and timing. The overall risk of publication bias was evaluated using Deeks’ funnel plot asymmetry test for diagnostic test accuracy meta-analyses applied to diagnostic odds ratios (DORs).

## 4. Results

### 4.1. Study Selection and Flow

**Figure 1:**
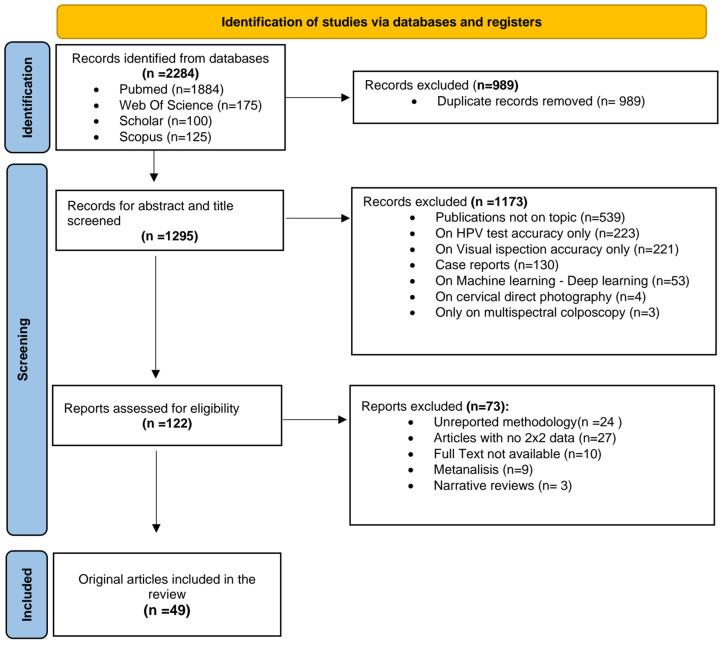
PRISMA-DTA flow diagram for study selection.

Using the PRISMA-DTA methodology, an initial 2284 articles were identified in the database search. After removing 989 duplicates, 1259 abstracts were screened. Applying inclusion and exclusion criteria, we conducted a full-text review of 122 studies, resulting in the final inclusion of 49 articles (Figure 1).

### 4.2. Characteristics of Included Studies and Populations

Forty-nine studies were included in the final analysis, representing a diverse geographic and methodological spectrum. The mean age of the patients was 35 years ± 5.73. The most represented country was China, contributing 18.4% of the included studies (n = 9), followed by the USA (14.2%, n = 7), Italy (12.2%, n = 6), and the UK (8.1%, n = 4). Other contributing countries included Canada (4.1%, n = 2), Iran (6.1%, n = 3), India (6.1%, n = 3), Brazil (4.0%, n = 2), Spain (4.1%, n = 2), the Netherlands (4.1%, n = 2), Denmark (4.1%, n = 2), and Germany (4.1%, n = 2). Single-study contributions came from Greece, Japan, Romania, Sweden, and Thailand. The countries were classified by income level according to World Bank country classifications (https://blogs.worldbank.org/) by income level for 2024–2025. In our study, we included 31 HICs (63.3%), 3 LICs (6.1%) and 15 MICs (30.6%). Regarding study design, the sample was nearly evenly divided between prospective (P) and retrospective (R) studies. Prospective studies comprised 46.9% (n = 23), while retrospective studies accounted for 53.1% (n = 26). The index text was standard colposcopy in every study. In five studies, the authors used dynamic spectral colposcopy, but we extracted data from the normal colposcope. The reference standard was biopsy (B), used in 100% of the cases (n = 49). Some studies also used a loop electrosurgical excision procedure after targeted biopsy (LEEP, L, 10.2% (n = 5), and hysterectomy (H) was used in a single study (2.0%) but those metrics was not included in the final analysis. A total of 77.6% (n = 38) of studies utilized single biopsy, while 22.4% (n = 11) multiple biopsies. All studies were set in second-level centers after positive screening. The studies’ characteristics are detailed in Table 1.

### 4.3. Pooled Analysis of Diagnostic Accuracy

**Figure 2:**
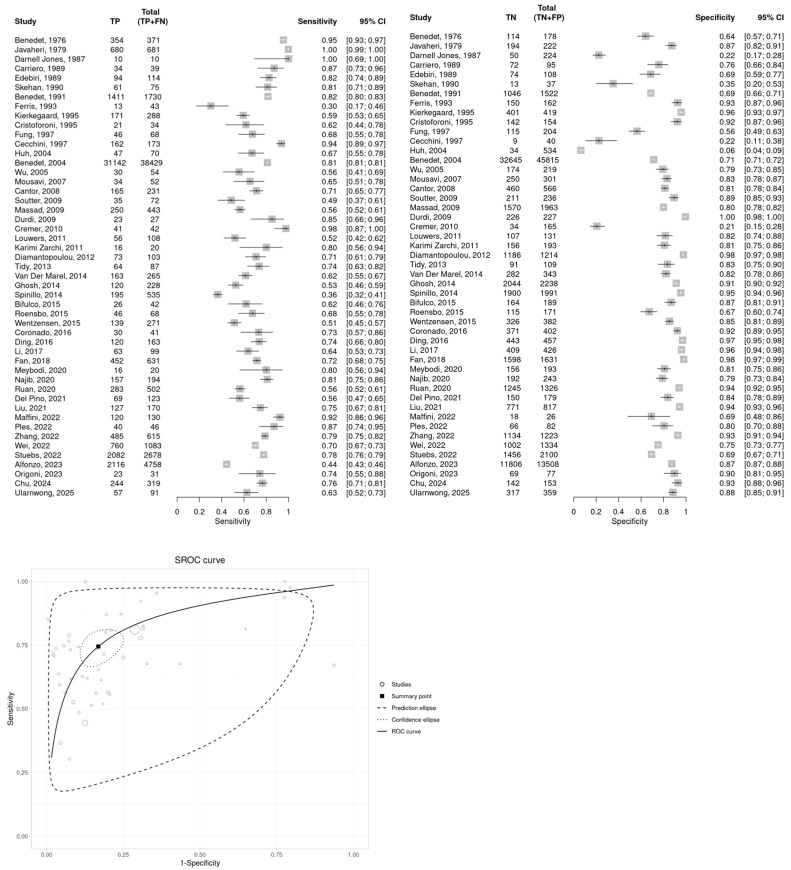
Funnel plot and sROC curve of the main analysis.

A total of 49 studies, including 141,355 colposcopic examinations, were included in the final analysis. The overall sensitivity was 0.745 (95% CI: 0.684–0.797) and specificity was 0.831 (95% CI: 0.770–0.879) (Figure 2). The diagnostic odds ratio was 14.388 (95% CI: 9.673–21.400). The positive likelihood ratio was 4.419 (95% CI: 3.260–5.991). Conversely, the negative likelihood ratio was 0.307 (95% CI: 0.249–0.378). The area under the curve was 0.859. Despite the favorable diagnostic metrics, significant heterogeneity was observed across studies. The bivariate random-effect meta-analysis showed substantial between-study heterogeneity in diagnostic accuracy with estimated variances in the logit-transformed sensitivity and specificity of 1.041 and 1.875, respectively. The median odds ratios were 2.65 and 3.69 for sensitivity and specificity, respectively. Overall heterogeneity was high, with a bivariate I^2^ of 93.2% with 95% prediction ellipse wide (area = 0.521). The tabular results of the pooled analysis of colposcopy’s diagnostic are presented in Table 2.

**Figure 3:**
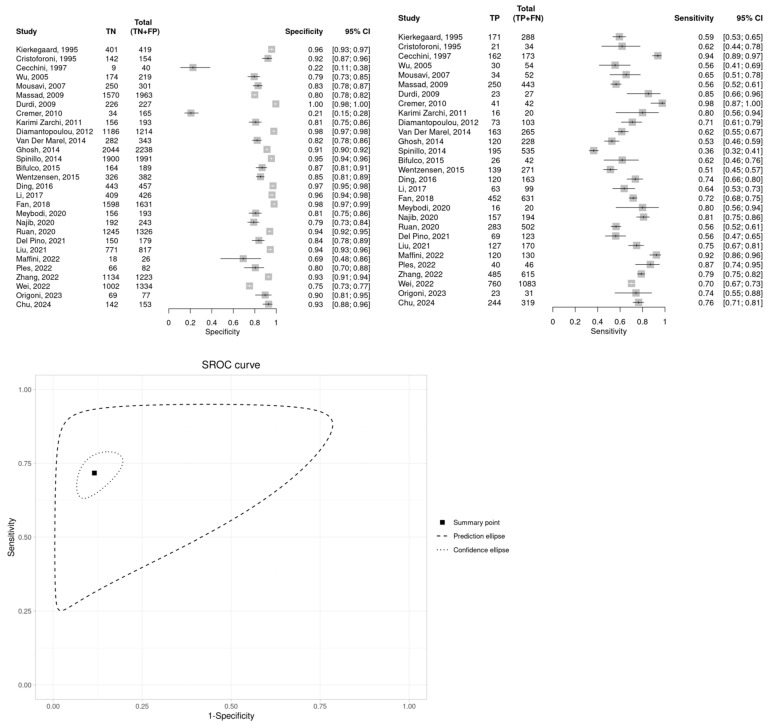
Subgroup analysis—estimate overall analysis of colposcopy’s diagnostic.

We performed a leave-out analysis excluding older studies (<1995), large cohorts (Alfonzo et Benedect) [15,59], high-risk-of-bias studies (Fung, 1997 Louwers, 2011, Stuebs, 2022, Ularnwong, 2025) [24,35,58,61] and studies including spectral analysis (Huh, 2004, Cantor, 2008 Soutter, 2009 Tidy, 2013 Roensbo, 2015 Coronado, 2016) [26,30,31,38,43,45]. In the final sub-analysis, we included 29 studies. The results showed an overall reduction in heterogeneity (bivariate i2 = 0.897) with an overall sensitivity of 0.717 (95% ci: 0.653–0.773) and specificity of 0.884 (95% ci: 0.827–0.924). The diagnostic odds ratio was 19.271 (95% ci: 12.763–29.097). The positive likelihood ratio was 6.174 (95% ci: 4.227–9.019). Conversely, the negative likelihood ratio was 0.116 (95% ci: 0.076–0.173). The area under the curve was 0.830. The results are shown in Figure 3.

### 4.4. Meta-Regressions for Moderators

In high-income countries (HICs), pooled sensitivity and specificity were 0.74 (95% CI 0.65–0.82) and 0.79 (95% CI 0.70–0.85), respectively. In contrast, low- and middle-income countries (LMICs) demonstrated similar sensitivity (0.75, 95% CI 0.68–0.80) but higher specificity (0.89, 95% CI 0.82–0.94). Correspondingly, the DOR was substantially higher in LMICs (24.01 vs. 10.54), with higher LR+ (6.86 vs. 3.46) and lower LR− (0.29 vs. 0.33). Meta-regression showed no significant difference in relative sensitivity between HICs and LMICs (*p* = 0.794), but relative specificity was significantly higher in LMICs (relative specificity 1.14, 95% CI 1.01–1.28; *p* = 0.043). The global test comparison was not significant (*p* = 0.071).

Prospective studies showed a pooled sensitivity of 0.72 (95% CI 0.63–0.79) and specificity of 0.83 (95% CI 0.73–0.90), while retrospective studies demonstrated slightly higher sensitivity (0.77, 95% CI 0.68–0.84) with similar specificity (0.83, 95% CI 0.75–0.89). Diagnostic accuracy was modestly higher in retrospective studies (DOR 16.42 vs. 12.25), with a lower LR− (0.28 vs. 0.34). However, meta-regression showed no significant differences in either relative sensitivity (*p* = 0.436) or relative specificity (*p* = 0.936) between prospective and retrospective studies. The global test comparison was not significant (*p* = 0.687). 

According to the pretest knowledge of patient’s HPV status by the colposcopists, we selected only a subgroup of studies published after the introduction of HPV screening test (n = 45,125), using as a threshold the year 2010. Studies incorporating HPV pre-testing demonstrated lower sensitivity (0.68, 95% CI 0.63–0.73) but markedly higher specificity (0.93, 95% CI 0.90–0.95) compared with studies without HPV pre-testing (sensitivity 0.73, 95% CI 0.61–0.82; specificity 0.79, 95% CI 0.70–0.86). HPV pre-testing was associated with substantially improved diagnostic performance, reflected by a higher DOR (28.76 vs. 10.24), higher LR+ (9.87 vs. 3.50), and a lower false-positive rate (0.07 vs. 0.21). Meta-regression confirmed a significantly higher relative specificity in studies with HPV pre-testing (relative specificity 1.17, 95% CI 1.06–1.29; *p* = 0.001), while relative sensitivity did not differ significantly (*p* = 0.50). The global test comparison was statistically significant (*p* = 0.001).

### 4.5. Results of Assessment of the Risk of Bias (RoB) and Publications’ Bias

**Figure 4:**
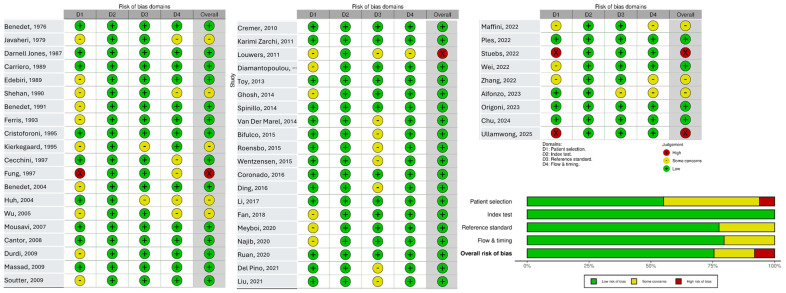
Results of assessment of risk of bias.

In total, 37 papers (75%) were judged as being at low RoB, 8 papers (16%) with some concerns of RoB and 4 papers (8%) as being at high RoB. The data are reported in Figure 2. Patient selection represented the main source of potential bias across included studies. While just over half of studies were judged to be at low risk, a substantial proportion had some concerns or high risk of bias. These issues raise the possibility of spectrum bias, which may have influenced pooled sensitivity and specificity estimates and contributed to the between-study heterogeneity observed in subgroup and meta-regression analyses. Only four studies were judged as being at high RoB because of concerns regarding the technique of execution of colposcopy and reporting and one for multiple exclusion of patients without pertinent reasons or some problems on the study flow. The results are presented in Figure 4. Deek’s funnel plot test for diagnostic odds ratios showed no significant risk of publication bias, with a result of t = −1.53, df = 31, a *p*-value = 0.1369 and a bias estimate of −8.8214 (SE = 5.7764). However, the test confirmed a high heterogeneity between studies, with τ^2^ = 121.76.

## 5. Discussion

This meta-analysis estimates the diagnostic accuracy of the colposcopic impression of CIN2+ lesions of the cervix across 49 high-quality studies, demonstrating moderate-to-high diagnostic accuracy. The overall sensitivity and specificity suggest that the test correctly identifies approximately 75% of true positives and 83% of true negatives across the included studies. The DOR is indicativeof test performance combining sensitivity and specificity, for which results greater above 10 suggest good diagnostic performance. PLR and NLR were, respectively, 4.33 and 0.30, indicating that a positive test multiplies the “odds of disease” by more than fourfold, and a negative test result reduces the “odds of disease” by nearly two-thirds. These likelihood ratios further support the clinical utility of colposcopy, especially in settings where ruling in or ruling out disease is critical. The diagnostic accuracy of colposcopy is high, with an AUC from sROC 0.8569. Despite the favorable diagnostic metrics, the heterogeneity across studies with high I^2^ and τ^2^ statistics indicates a substantial variability among studies that is difficult to be attributed to chance alone. Taken together, while the diagnostic test shows promising accuracy, the high heterogeneity suggests caution in interpreting these results. Meta-regression analyses identified several study-level factors that partially explained heterogeneity in diagnostic performance. The pre-testing knowledge of patient’s HPV status emerged as the most influential modifier, showing a robust and statistically significant improvement in specificity and overall diagnostic accuracy. This moderator contributes to triage the tested population for clinically relevant disease, thus reducing false-positive findings. The observed increase in specificity and slight reduction in sensitivity with HPV pre-testing may reflect a change in clinical decision-making during colposcopy. A negative HPV testing may be reassuring for the clinicians, leading to a more conservative approach and to miss low-grade or early lesions. Although, awareness of a positive HPV result may change the clinician’s threshold for biopsy, differently interpreting borderline findings. Overall, these findings suggest that the knowledge of the patient’s pre-test HPV status modifies colposcopist’s clinical decision, improving efficiency at the cost of a small reduction in lesion detection. This pattern is consistent with a diagnostic threshold shift and possible confirmation bias. The analysis of geographic setting also influenced test performance, with studies from low- and middle-income countries demonstrating higher specificity than those from high-income countries, while sensitivity remained comparable; this may be related to spectrum bias due to relative increase in high-grade and advanced lesion’s prevalence in low–middle income countries where the screening strategies are less effective and there is a higher probability of detecting more advanced or clinically evident high-grade lesions among women referred for colposcopyIn contrast, study design (prospective vs. retrospective) did not significantly affect sensitivity or specificity, suggesting that methodological differences related to study design were not major drivers of heterogeneity in this analysis. Overall, these results indicate that diagnostic accuracy is more strongly influenced by clinical context and testing pathways than by study design alone, underscoring the importance of considering screening strategies and population characteristics when interpreting pooled estimates. Subgroup analyses or meta-regression may be warranted to explore potential sources of variability and to better define the contexts in which this test performs optimally. An important consideration is the risk of bias across the included studies, which may have impacted the results. Based on the QUADAS-2 assessment, eight papers showed some concerns, and four were at high RoB, particularly in the index test and reference standard domains. These limitations highlight the need for cautious interpretation and underscore the importance of rigorous methodological quality in future research.

The present study has several limitations that should be considered when interpreting the findings. First, the high heterogeneity among the included studies substantially limits the reliability and generalizability of the pooled estimates. To address this issue, we performed subgroup analyses, which reduced heterogeneity without substantially altering the overall results concerning colposcopic diagnostic accuracy, which remains high.

Factors contributing significantly to the heterogeneity are the inclusion of studies with different colposcopic criteria, examination protocols, diagnostic thresholds, and examiner experience. Moreover, differences in patient populations, screening settings, and disease prevalence may also have affected the pooled sensitivity and specificity estimates. However, the inclusion criteria target was to enhance study homogeneity including only second-level referral centers, with expert trained colposcopists and use of standardized terminology, excluding studies with significant methodological inconsistencies. The choice to cover a wide geographical setting, including studies from different countries and healthcare systems, may also limit the applicability of the findings to specific clinical settings; however, this characteristic may contribute to give a panoramic view of the overall colposcopic accuracy worldwide based on real-world data. The choice to include older studies may not fully reflect the current clinical practice. Nonetheless, subgroup analyses excluding those confirmed the main findings while showing lower heterogeneity, supporting the robustness of the results. Meta-regression was difficult to carry out because of the high heterogeneity and lack of adjunctive clinical data in the original papers. However, HPV testing results was consistently reported in the major of the most recent included studies. Finally, this meta-analysis focused exclusively on diagnostic accuracy and did not evaluate clinically relevant outcomes such as disease progression, treatment decisions, long-term follow-up, or patient management, which are essential for assessing the overall clinical impact of colposcopy. Further studies are needed to evaluate those data.

## 6. Conclusions

Our results are the first to assess the diagnostic accuracy of colposcopy in a large, multicenter and international cohort. Colposcopy is confirmed as a diagnostic method for detection and clinical evaluation of oncological pathology of the cervix in second-level settings. However, our data highlight that its diagnostic performance may be strongly influenced by the expertise of operators, in particular by the knowledge of pre-test information and by the clinical settings. When these elements are integrated thoughtfully into the clinical assessment, the pre-test assessment of CIN2+ is more accurately defined, allowing colposcopy to be used more effectively for risk stratification and decision-making. This meta-analysis demonstrates that, despite the traditional belief that colposcopy is inherently subjective and operator-dependent, its diagnostic accuracy is high for CIN2+ lesions. Given that more robust data are needed, clinicians should interpret test results considering pre-test probabilities, local settings, and the quality of the evidence. Emerging AI-driven Machine Learning tools have the potential to improve lesion detection, standardize assessment criteria, and reduce inter-observer variability. Such tools could also facilitate training for less-experienced clinicians and support more consistent clinical decision-making across diverse healthcare settings. Further research is needed to validate AI algorithms in large, diverse populations and to assess their impact on clinical outcomes, cost effectiveness, and integration into routine cervical cancer screening and triage pathways. The fusion of a detailed assessment of the patient’s clinical history with the conventional colposcopic impression and emerging technological innovations may representa promising future direction for achieving a more accurate, personalized, and efficient colposcopic and clinical evaluation of CIN2+ lesions.

## Figures and Tables

**Table 1:** Main characteristics of included studies.

Author, Year	Nation	Numerosity	Sensitivity	Specificity	PPV	NPV	AUC	HPV Status Pre-Test?	Income	Study Design
Benedet, 1976 [15]	Canada	549	0.95	0.64	0.85	0.87	0.85	N	H	P
Javaheri, 1979 [16]	USA	903	1.00	0.87	0.96	0.99	0.97	N	H	R
Darnell, Jones 1987 [17]	USA	234	1.00	0.22	0.05	1.00	0.26	N	H	P
Carriero, 1989 [18]	Italy	134	0.87	0.76	0.60	0.94	0.79	Y	H	R
Edebiri, 1989 [19]	UK	222	0.82	0.69	0.73	0.79	0.76	N	H	R
Skehan, 1990 [20]	UK	112	0.81	0.35	0.72	0.48	0.66	N	H	R
Benedet, 1991 [21]	Canada	3252	0.82	0.69	0.75	0.77	0.76	N	H	R
Kierkegaard, 1995 [22]	Denmark	707	0.59	0.96	0.90	0.77	0.81	N	H	R
Cristoforoni, 1995 [23]	Italy	188	0.62	0.92	0.64	0.92	0.87	N	H	P
Fung, 1997 [24]	Hong Kong	94	0.68	0.56	0.34	0.84	0.59	Y	H	R
Cecchini, 1997 [25]	Italy	213	0.94	0.23	0.84	0.45	0.80	N	H	R
Huh, 2004 [26]	USA	604	0.67	0.06	0.09	0.60	0.13	N	H	P
Benedet, 2004 [27]	Canada	84,244	0.81	0.71	0.70	0.82	0.76	N	H	R
Wu, 2005 [28]	China	273	0.56	0.79	0.40	0.88	0.75	Y	M	R
Mousavi, 2007 [29]	Iran	353	0.65	0.83	0.40	0.93	0.80	N	M	P
Cantor, 2008 [30]	USA, Canada	797	0.71	0.81	0.61	0.87	0.78	Y	H	P
Soutter, 2009 [31]	UK, Greece	308	0.49	0.89	0.58	0.85	0.80	N	H	P
Massad, 2009 [32]	USA	2406	0.56	0.80	0.39	0.89	0.76	N	H	P
Durdi, 2009 [33]	India	254	0.85	1.00	0.96	0.98	0.98	N	L	P
Cremer, 2010 [34]	USA	207	0.98	0.21	0.24	0.97	0.36	N	M	R
Louwers, 2011 [35]	Netherlands	239	0.52	0.82	0.70	0.67	0.68	N	H	P
Karimi, Zarchi 2011 [36]	Iran	213	0.80	0.81	0.30	0.98	0.81	N	M	P
Diamantopoulou, 2012 [37]	Greece	1317	0.71	0.98	0.72	0.98	0.96	Y	H	P
Tidy 2013 [38]	UK, IRL	196	0.74	0.83	0.78	0.80	0.79	N	H	P
Van Der Marel, 2014 [39]	Netherlands	608	0.62	0.82	0.73	0.73	0.73	Y	H	P
Ghosh, 2014 [40]	India	2466	0.53	0.91	0.38	0.95	0.88	Y	L	R
Spinillo, 2014 [41]	Italy	2526	0.36	0.95	0.68	0.85	0.83	N	H	P
Bifulco, 2015 [42]	Italy	231	0.62	0.87	0.51	0.91	0.82	N	H	P
Roensbo, 2015 [43]	Denmark	239	0.68	0.67	0.45	0.84	0.67	N	H	R
Wentzensen, 2015 [44]	USA	653	0.51	0.85	0.71	0.71	0.71	Y	H	P
Coronado, 2016 [45]	Spain	443	0.73	0.92	0.49	0.97	0.91	Y	H	P
Ding, 2016 [46]	China	620	0.74	0.97	0.90	0.91	0.91	Y	M	R
Li, 2017 [47]	China	525	0.64	0.96	0.79	0.92	0.90	Y	M	R
Fan, 2018 [48]	China	2262	0.72	0.98	0.93	0.90	0.91	Y	M	R
Meybodi, 2020 [49]	Iran	213	0.80	0.81	0.30	0.98	0.80	N	M	P
Najib, 2020 [50]	India	437	0.81	0.79	0.75	0.84	0.80	N	L	P
Ruan, 2020 [51]	China	1828	0.56	0.94	0.78	0.85	0.84	Y	M	R
Del Pino, 2021 [52]	Spain	302	0.56	0.84	0.70	0.74	0.73	N	H	R
Liu, 2021 [53]	China	987	0.75	0.94	0.73	0.95	0.91	Y	M	R
Maffini, 2022 [54]	Brazil	156	0.92	0.69	0.94	0.64	0.88	N	M	P
Ples, 2022 [55]	Romania	128	0.87	0.80	0.71	0.92	0.83	N	H	P
Zhang, 2022 [56]	China	1838	0.79	0.93	0.84	0.90	0.88	Y	M	R
Wei, 2022 [57]	China	2417	0.70	0.75	0.70	0.76	0.73	Y	M	R
Stuebs, 2022 [58]	Germany	4778	0.78	0.69	0.76	0.71	0.74	N	H	R
Alfonzo, 2023 [59]	Sweden	18,266	0.44	0.87	0.55	0.82	0.76	N	H	P
Origoni, 2023 [5]	Italy	100	0.73	0.87	0.74	0.90	0.84	Y	H	R
Chu, 2024 [60]	China	512	0.68	0.93	0.96	0.65	0.86	Y	M	R
Ularnwong, 2025 [61]	Thailand	450	0.62	0.88	0.50	0.90	0.80	N	M	R

HPV Statut pretest: N: No, Y: Yes. Income: H: High Income Country; M: Medium-income Country; L: Low-income country. Study design: P: prospetive; R: retrospective.

**Table 2:** Estimate overall analysis of colposcopy’s diagnostic.

Summary Statistics	Estimate	95% LCI	95% UCI
Sensitivity	0.745	0.684	0.797
Specificity	0.831	0.77	0.879
DOR	14.388	9.673	21.4
LR+	4.419	3.26	5.991
LR−	0.307	0.249	0.378
FPR	0.169	0.121	0.23
**Additional statistics**	**Estimate**		
Logit sensitivity	1.07		
Logit specificity	1.596		
Var (logit(sen))	1.041		
Var (logit(spec))	1.875		
SE (logit(sens))	0.152		
SE (logit(spec))	0.198		
Covariance (ES)	−0.011		
Correlation	−0.374		
**Heterogeneity assessment**	**Estimate**		
Var logit(sen)	1.041		
Var logit(spe)	1.875		
MOR sensitivity	2.646		
MOR specificity	3.692		
Bivariate I^2^	0.932		
Area 95% Prediction Ellipse	0.521		

## Data Availability

All the data are publicity available and included in this article.

## Supplementary Materials

The following supporting information can be downloaded at: https://www.mdpi.com/article/10.3390/cancers18142239/s1, Supplementary File S1: PRISMA Checklist.

## Abbreviations

The following abbreviations are used in this manuscript:

cervical intraepithelial neoplasia grade 2 or higher	CIN2+
cervical cancer	CC
human papillomavirus	HPV
summary receiver operating characteristic	sROC
Preferred Reporting Items for Systematic Reviews and Meta-Analyses	PRISMA
artificial intelligence	IA
Standards for Reporting Diagnostic Accuracy	STARD
positive and negative likelihood ratios	PLR and NLR
diagnostic odds ratios	DOR
confidence interval	CI
area under the curve	AUC
standard error	SE
Quality Assessment of Diagnostic Accuracy Studies	QUADAS-2
